# Impact of Multiple Sclerosis Risk Polymorphism rs7665090 on MANBA Activity, Lysosomal Endocytosis, and Lymphocyte Activation

**DOI:** 10.3390/ijms23158116

**Published:** 2022-07-23

**Authors:** Adela González-Jiménez, Pilar López-Cotarelo, Teresa Agudo-Jiménez, Ignacio Casanova, Carlos López de Silanes, Ángeles Martín-Requero, Fuencisla Matesanz, Elena Urcelay, Laura Espino-Paisán

**Affiliations:** 1 Laboratorio de Genética de Enfermedades Complejas, Instituto de Investigación Sanitaria del Hospital Clínico San Carlos (IdISSC), 28040 Madrid, Spain; adela.gonzalez.jimenez@salud.madrid.org (A.G.-J.); pilar.lopezcotarelo@salud.madrid.org (P.L.-C.); tere844.taj@gmail.com (T.A.-J.); elena.urcelay@salud.madrid.org (E.U.); 2 Servicio de Neurología, Hospital Universitario de Torrejón, 28850 Madrid, Spain; i.casanovap@gmail.com (I.C.); carlosdesilanes@yahoo.es (C.L.d.S.); 3 Centro de Investigaciones Biológicas, CSIC, 28006 Madrid, Spain; amrequero@cib.csic.es; 4 Centro de Investigación Biomédica en Red de Enfermedades Neurodegenerativas (CIBERNED), Instituto Carlos III, 28029 Madrid, Spain; 5 Departmento Biología Celular e Inmunología, Instituto de Parasitología y Biomedicina López Neyra, IPBLN-CSIC, 18016 Granada, Spain; lindo@ipb.csic.es

**Keywords:** multiple sclerosis, MANBA, immune system, T cells, B cells, immunoregulation, genetic biomarkers

## Abstract

Deficiencies in Mannosidase β (MANBA) are associated with neurological abnormalities and recurrent infections. The single nucleotide polymorphism located in the 3′UTR of *MANBA*, rs7665090, was found to be associated with multiple sclerosis (MS) susceptibility. We aimed to study the functional impact of this polymorphism in lymphocytes isolated from MS patients and healthy controls. A total of 152 MS patients and 112 controls were genotyped for rs7665090. *MANBA* mRNA expression was quantified through qPCR and MANBA enzymatic activity was analyzed. Upon phytohemagglutinin stimulation, immune activation was evaluated by flow cytometry detection of CD69, endocytic function, and metabolic rates with Seahorse XFp Analyzer, and results were stratified by variation in rs7665090. A significantly reduced gene expression (*p* < 0.0001) and enzymatic activity (*p* = 0.018) of MANBA were found in lymphocytes of MS patients compared to those of controls. The rs7665090*GG genotype led to a significant β-mannosidase enzymatic deficiency correlated with lysosomal dysfunction, as well as decreased metabolic activation in lymphocytes of MS patients compared to those of rs7665090*GG controls. In contrast, lymphocytes of MS patients and controls carrying the homozygous AA genotype behaved similarly. Our work provides new evidence highlighting the impact of the MS-risk variant, rs7665090, and the role of *MANBA* in the immunopathology of MS.

## 1. Introduction

Multiple Sclerosis (MS) is a chronic, inflammatory, and demyelinating disease of autoimmune origin and one of the primary neurological causes of physical disability in young adults [1]. The etiology of this complex disease is still elusive, but the influence of both genetic and environmental factors has been described [2]. The genetic architecture of MS was partially established through genome-wide association studies (GWAS), which unveiled 233 risk single nucleotide polymorphisms (SNPs), accounting for barely half of the MS heritability [3]. Most of these MS susceptibility polymorphisms map to non-coding regulatory regions of the genome and their functional impact needs to be characterized. Recently, several comprehensive analyses have been reported integrating GWAS results in different traits with transcriptomic, epigenomic, and proteomic data through computational workflows [4,5,6,7,8] and have generated resources to advance the understanding of the mechanisms predisposing to MS and other immune-mediated diseases. These multi-omic efforts evidence that the risk SNPs frequently act through regulatory mechanisms affecting the expression of neighbor genes in *cis*, but they may also act over long distances across the entire genome, in *trans*. Nevertheless, the exact causal alleles and mechanisms that underlie associations of genetic variants to disease have remained largely unknown, hindering translational results. The ascertainment of the genes involved in MS susceptibility facilitates the formulation of the causal mechanisms behind this complex immune-mediated condition, and it has proven an extremely difficult task. Beyond this gene prioritization, subsequent functional studies are required to expand the knowledge of gene targets in disease-relevant cell types contributing to disease onset, which may provide opportunities for drug development or repurposing [9,10].

Efforts to unveil the plausible etiological genes corresponding to the identified GWAS polymorphisms, trying to understand how those MS-associated variants affect the expression and the function of single genes, have often times yielded successful outcomes [11,12]. In the present work, we aimed to investigate one of these MS risk SNPs, rs7665090, located in chromosome 4q22-25 in proximity to the *MANBA* (*Mannosidase beta*) gene (OR= 1.09, risk allele A) [13,14]. The MANBA protein belongs to the glycosyl hydrolase 2 family and catalyzes the hydrolysis of terminal, non-reducing β-D-mannose residues found in N-linked oligosaccharides of glycoproteins. Localized in the lysosome, it participates in the final steps of N-linked-glycan degradation [15]. Mutations in *MANBA* resulting in deficient activity lead to lysosomal accumulation of disaccharides and the development of β-mannosidosis, a very rare disease with few reported cases [16,17]. β-mannosidosis is a lysosomal storage disease promoting a wide spectrum of neurological abnormalities, behavioral disturbances, hearing loss, and recurrent infections [18,19,20]. In addition, genetic variants in *MANBA* have also been identified in infantile nystagmus [21] and attention deficit hyperactivity disorder [22]. MANBA has also been implicated in neutrophil degranulation [23,24], and the mentioned MS-risk polymorphism has been shown to distort the balance of allele-specific expression in lymphocyte subtypes of interest in MS pathogenesis [25]. Therefore, MANBA seems to play a role both in the immune response and in correct neurological function.

The analysis of the MS risk polymorphisms established by the largest GWAS performed to date [4] stressed the importance of peripheral immune cells of many different types in MS susceptibility, not only of T cells but also of B cells, whose role in MS has been more recently unveiled [26], as well as natural killer and dendritic cells which displayed strong enrichment of MS susceptibility genes. In fact, as recently reported, key disease processes formerly attributed to a single cell type appear to be more widespread than previously recognized, reflecting the intrinsic redundancy of the immune system [27]. In the present work, we aimed to compare immune cells from MS patients and healthy controls to gain a deeper understanding of the mechanisms underlying the role of rs7665090 and the *MANBA* gene in its pathogenesis, which ultimately could improve clinical outcomes.

## 2. Materials and Methods

### 2.1. Study Population/Patients and Controls

The study included a total of 152 MS patients (60.3% females) and 112 healthy controls (63.3% females), all Caucasian and with mean ages of 42 ± 13 and 43 ± 11 years, respectively. Participants were recruited from hospitals in the Madrid metropolitan area during routine revisions in their Neurology departments. Patients were diagnosed with relapsing-remitting multiple sclerosis (RRMS) according to McDonald’s criteria [28]; all of them were under long-term treatment with interferon-β formulations or glatiramer acetate and had no evidence of relapse before or after the extraction. None of the control subjects reported first or second-degree relatives with any immune-mediated disease. All subjects were recruited after written informed consent and the study received approval from the Ethics Committee from Hospital Clínico San Carlos (CE16/211-E and CE20/740-E_BC).

### 2.2. Cell and DNA Extraction

Peripheral blood samples were collected and peripheral blood mononuclear cells (PBMCs) were separated with Lymphoprep (07851, Stemcell Technologies, Vancouver, BC, Canada). Briefly, 10 mL of whole blood was diluted in 10 mL of Hank’s balanced salt solution (HBSS, 21-022-CV, Corning, NY, USA) and pipetted slowly over 10 mL of Lymphoprep in a 50 mL tube. The gradient solution was centrifuged 20 min at 900× *g* and room temperature without break. Afterward, PBMCs were collected with a Pasteur pipette, washed with HBSS, and counted in the Neubauer chamber. Subsequently, vials containing 10^7^ PBMCs in freezing medium, consistent with fetal bovine serum (FBS, F9665, Sigma Aldrich, St. Louis, MO, USA) with 10% DMSO (D5879, Sigma Aldrich, Bremen, Germany), were preserved in liquid nitrogen until further analysis.

Genomic DNA was extracted from the granulocyte phase of the density gradient following a salting-out procedure. Cells were washed two times with a hypoosmotic buffer (sucrose 0.32 mM, MgCl_2_ 0.005 mM, Tris-HCl 1 M, and Triton X-100 1%). Pellets in 5 mL of buffer B (NaCl 0.075 mM and EDTA 0.024 M), 20% SDS and 25 μg proteinase K were left overnight at 51 °C. The next day, 1.75 mL of a saturated 6 M NaCl solution was added to the sample and centrifuged at 3000 rpm for 30 min to precipitate proteins. The supernatant was transferred to a tube and DNA was precipitated with an equal volume of isopropanol, captured with a glass pipette, washed in 70% ethanol, and finally suspended in 500 μL TE buffer, quantified, and preserved at −20 °C.

### 2.3. Genotyping

A total of 152 MS patients and 112 healthy controls were genotyped for rs7665090 by TaqMan technology in a 7900HT Fast Real-Time PCR System (Applied Biosystems, Waltham, MA, USA) following the manufacturer’s protocol with slight modifications. Briefly, 30 ng of genomic DNA was mixed with a master mix containing 0.045 μL of the rs7665090 probe (C___1679293_10), 1.3 μL of Genotyping master mix (4371355, Applied Biosystems, Waltham, MA, USA) and 2.5 μL distilled water to a total volume reaction of 5 μL. The mix was run in an RT-PCR HT7900 equipment with the following protocol: 2 min, 50 °C for amperase activation, 10 min at 95 °C for DNA denaturation, and 40 cycles of denaturation and synthesis (15 s at 92 °C for denaturation of DNA and 1 min at 60 °C for hybridization and synthesis). Genotypes were obtained through the allelic discrimination protocol and confirmed individually by checking the qPCR amplification plots.

### 2.4. Gene Expression

Total RNA was isolated from PBMCs of 152 MS patients and 112 healthy controls with Trizol reagent (15596018, Invitrogen, Carlsbad, CA, USA), following the manufacturer’s protocol, quantified in a NanoDrop ND-1000 spectrophotometer (NanoDrop Products; Wilmington, DE, USA), and reverse transcribed to cDNA using the High capacity RNA-to-cDNA kit (4387406, Applied Biosystems, Waltham, MA, USA). Then, it was analyzed with a 7900HT Fast Real-Time PCR System (Applied Biosystems, Waltham, MA, USA). *GUSB* was selected as a housekeeping gene. The reaction consisted of 5 μL TaqMan Gene expression master mix (4369016, Applied Biosystems, Waltham, MA, USA), 0.5 μL of *MANBA* expression probe 20X (Hs01099178), 0.5 μL of *GUSB* probe as a housekeeping gene (4326320E), 2 μL of distilled water and 2 μL of the sample cDNA. The RT-PCR conditions were the same as described for genotyping in Section 2.3. Taqman probes for *MANBA* and *GUSB* were purchased from Applied Biosystems. Data were analyzed with DataAssist v3.01 software (Applied Biosystems, Waltham, MA, USA).

### 2.5. Assessment of β-Mannosidose Activity 

PBMCs from 30 healthy controls and 47 MS patients were lysed and homogenized by sonication in distilled water. Protein concentration was quantified by Bradford assay using BSA (bovine serum albumin) as standard protein (Pierce Biotechnology, Rockford, IL, USA), and the activity assay was performed according to Yu et al. protocol [21], with small modifications. Briefly, the MANBA substrate 4-MU-β-mannosidose (4-Methylumbelliferyl-β-mannoside, M0905, Sigma, St Louis, MO, USA) was prepared in 0.2 M NaHPO_4_/0.1 M Citric Acid, pH 4.2, to a final concentration of 2 mM. Subsequently, 2 μg of protein lysates (10 μL) were incubated with 20 μL of 4-MU-β-mannosidose for 1 h at 37 °C. The reaction was stopped by adding 200 μL of 0.5 M Na_2_CO_3_, pH 10.7, and activity was assessed by measuring the fluorescence of the reaction product (4-MU; excitation 355 nm, emission 460 nm) with a Varioscan Flash 40052 Microplate reader.

### 2.6. Cell Activation

PBMCs (3 × 10^5^) from 10 healthy controls and 15 MS patients were cultured without stimulus or stimulated with 10 μg/mL of phytohemagglutinin (PHA, L1668, Sigma-Aldrich, Bremen, Germany) in an RPMI medium supplemented with 10% FBS, 1% penicillin-streptomycin and 1% L-glutamine (Sigma Aldrich, Bremen, Germany). Activation was assessed 17 h later and cells were stained with anti-CD69-FITC (clone FN50, 310904), anti-CD3-PE (clone HIT3a, 300308), anti-CD20-APC (clone 2H7, 302310), and 7-AAD (420404) to exclude non-viable cells (Biolegend, San Diego, CA, USA). Briefly, cells were harvested, placed in cytometry tubes, and 2.5 μL of each monoclonal antibody was added. Samples incubated 30 min at 4 °C in the dark were subsequently washed and suspended in 300 μL PBS. Finally, 1.5 μL of the viability dye 7-AAD was added. Samples were analyzed in a Gallios flow cytometer (Beckman Coulter, Brea, CA, USA) and data were parsed with Kaluza 2.1 software (Beckman Coulter, Brea, CA, USA). Cells were gated as CD3^+^CD20^−^ (T cells), CD3^−^CD20^+^ (B cells), or CD3^−^CD20^−^ (mainly NK cells), and the gating strategy is depicted in Supplementary Figure S1. To evaluate the effect of PHA on both the percentage of activated cells and CD69 expression measured as mean fluorescence intensity (MFI) on each cell type, the interaction variable “MFI of CD69^+^ cells * increment of % of CD69^+^ cells” was generated and analyzed.

### 2.7. Endocytic Activity

For the evaluation of the endocytic activity, a maximum of 30 controls and 26 MS patients were selected. For each subject and test, samples unstimulated and stimulated with 10 μg/mL of PHA for 24 h were prepared, and the fold-change in endocytosis due to lymphocyte activation was calculated. For general fluid-phase endocytosis, 0.5 mg/mL of Dextran 10 Kda marked with Alexa fluor 488 (D22910, Invitrogen, Waltham, MA, USA) was added to 3 × 10^5^ PBMCs in RPMI supplemented with 10% FBS, 1% Penicillin-streptomycin and 1% L-glutamine, and left for 15 min at 37 °C with 5% CO_2_. Cells were subsequently harvested, washed four times (300 g, 10 min, 4 °C) in cold phosphate saline buffer (PBS) with 0.1% bovine serum albumin (BSA), stained with monoclonal antibodies anti-CD3 and anti-CD20, and with viability dye 7-AAD, according to the protocol described in Section 2.6, and acquired immediately in a CytoFLEX cytometer. For receptor-mediated endocytosis, 25 μg/mL of BSA marked with Alexa fluor 488 (A13100, Invitrogen, Waltham, MA, USA) was added to 3 × 10^5^ PBMCs cultured in supplemented RPMI, as described before, and left for 4 h at 37 °C with 5% CO_2_. After that, cells were washed 2 times (300 g, 10 min, 4 °C) with cold PBS, stained with monoclonal antibodies anti-CD3, anti-CD20, and viability dye 7-AAD, according to the protocol described in Section 2.6, and acquired immediately in a CytoFLEX cytometer (Beckman Coulter, Brea, CA, USA). Data were analyzed with Kaluza 2.1 software. The gating strategy is presented in Supplementary Figure S2.

### 2.8. Cell Metabolism

Oxygen Consumption Rate (OCR) and Extracellular Acidification Rate (ECAR) of 12 controls and 19 patients were assessed in a Seahorse XFp extracellular flux analyzer (Agilent Technologies, Santa Clara, CA, USA). For each participant, cells were stimulated with 5 μg/mL PHA for 24 h to evaluate the metabolism of activated PBMCs, and unstimulated samples were kept in parallel to test basal metabolism. Both conditions were assayed in triplicate by seeding 2 × 10^5^ PBMCs per well onto poly-D-lysine coated XFp plates (103022-100, Agilent Technologies, Santa Clara, CA, USA) for 45 min in XF DMEM medium pH 7.4 (103575-100, Agilent Technologies, Santa Clara, CA, USA) supplemented with glucose (10 mM) and pyruvate (2 mM). The mitochondrial and glycolytic functions were then assessed with the Cell MitoStress Test kit (103010-100, Agilent Technologies, Santa Clara, CA, USA), following the manufacturer’s instructions. Briefly, cells were treated over seventy minutes with mitochondrial oxidative phosphorylation (OXPHOS) selective inhibitors: 1.5 μM of oligomycin, an inhibitor of the ATP synthase; 0.5 μM FCCP (carbonylcyanide-p-trifluoromethoxyphenylhydrazone), a mitochondrial OXPHOS uncoupler, and a cocktail of rotenone and antimycin A at 0.5 μM, inhibitors of complex I and III from the respiratory chain. OCR and ECAR were registered during the run, allowing us to assess mitochondrial performance (OCR) and glycolytic function (ECAR before and after the addition of oligomycin).

### 2.9. Statistical Analysis

Normality was confirmed for all variables with the Kolmogorov–Smirnov test. Outliers were detected with the Grubbs’ test in the Graphpad online tool (https://www.graphpad.com/quickcalcs/Grubbs1.cfm, accessed on 20 May 2022) and comparisons were subsequently performed with Student’s *t*-test and ANOVA test; statistical analyses were performed with SPSS v15.0.1 (Chicago, IL, USA) and graphical representations with GraphPad Prism v9.0 (San Diego, CA, USA).

## 3. Results

### 3.1. Decreased MANBA Expression and Enzymatic Activity in Lymphocytes from MS Patients Compared to Those from Healthy Controls

**Figure 1:**
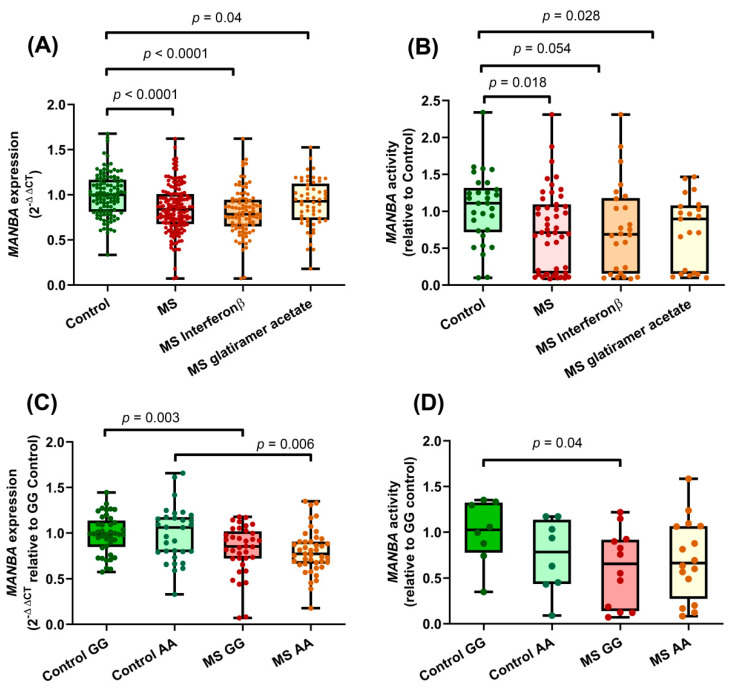
*MANBA* expression and activity in PBMCs. (**A**) *MANBA* expression in PBMCs from healthy donors and MS patients (controls *n* = 112; MS *n* = 151; interferon β *n* = 94; glatiramer acetate *n* = 57). Percentage of difference between means in Control vs. MS = 15.5 ± 3.13%; Control vs. interferon β = 19.31 ± 3.43%; Control vs. MS glatiramer acetate = 9.4 ± 4.0%. (**B**) MANBA activity in PBMCs from healthy donors and MS patients (controls *n* = 30; MS *n* = 47; interferon β *n* = 26; glatiramer acetate *n* = 21). Percentage of difference between means in Control vs. MS = 29.59 ± 12.2%; Control vs. MS interferon β = 28.61 ± 14.52%; Control vs. MS glatiramer acetate = 30.8 ± 13.64%. (**C**) *MANBA* expression in controls and MS patients stratified by rs7665090 genotypes (controls GG *n* = 41; controls AA *n* = 31; MS GG *n* = 36; MS AA *n* = 43). Percentage of difference between means in Control GG vs. MS GG= 16.84 ± 5.5%; Control AA vs. MS AA= 18.82 ± 6.36%. (**D**) Effect of rs7665090 genotypes on MANBA activity (controls GG *n* = 10; controls AA *n* = 9; MS GG *n* = 13; MS AA *n* = 14). Percentage of difference between means in Control GG vs. MS GG = 39.17 ± 17.7%. Box and whiskers diagrams: the line represents the median value, the box the 25 and 75 percentiles and whiskers mark maximum and minimum values. All comparisons were performed with Student’s *t* test.

PBMCS from MS patients displayed significantly lower levels of *MANBA* mRNA compared to those from healthy controls (*p* < 0.0001, Figure 1A). This difference persisted when patients were stratified according to their MS treatment: both β-interferon and glatiramer acetate treated patients exhibited significantly lower *MANBA* expression than controls.

We also tested the enzymatic activity in PBMCs lysates from MS patients and healthy controls. The activity was assessed by detecting the fluorescence signal generated when MANBA catalyzed the hydrolysis of non-fluorescent 4-methylumbellifryl-β-mannopyranoside into the fluorescent 4-methylumbellifryl. As shown in Figure 1B, PBMCs from MS patients presented lower β-mannosidase activity compared to those from healthy controls (*p* = 0.018), and again this difference was observed independently of the patient’s treatment.

The effect of the MS-risk polymorphism rs7665090 on *MANBA* expression was then examined. In the Iberian Spanish population, the rs7665090 alleles have very similar frequencies [29], being the G variant the major allele. Concordantly, in our sample, the rs7665090*G allele showed a frequency of 0.51. *MANBA* mRNA levels were analyzed after rs7665090 stratification in PBMCs from both MS patients and controls, and no allele-specific difference was observed (Figure 1C). Regarding the influence of extreme genotypes on β-mannosidase activity, a significant difference was found between PBMCs from homozygous rs7665090*GG controls with an increased activity compared to those from MS patients with the same genotype. In contrast, cells from patients and controls homozygous for the AA genotype displayed analogous levels of enzymatic activity (Figure 1D).

### 3.2. Endocytic Activity Is Modified by rs7665090

**Figure 2:**
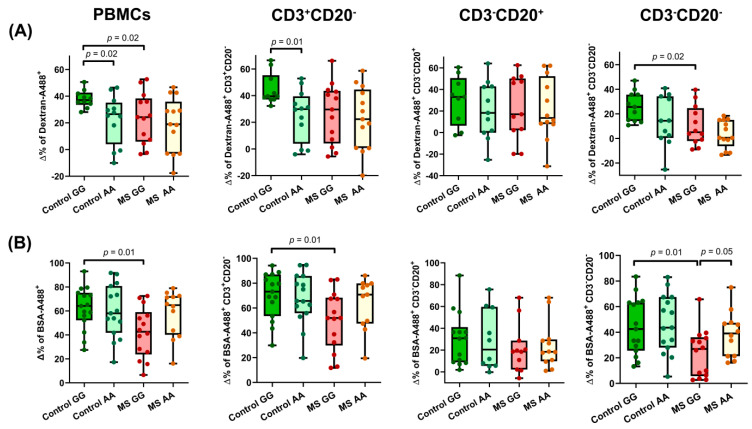
Effect of rs7665090 genotypes on endocytosisin PBMCs from healthy donors and MS patients pre-stimulated for 24 h or not with PHA. (**A**) Endocytosis of A488-Dextran 10 KDa at 0.5 mg/mL for 15 min by overall PBMCs, T cells (CD3^+^CD20^−^), B cells (CD3^−^CD20^+^) and other lymphocytes (CD3^−^CD20^−^) was assessed (controls GG *n* = 10; controls AA *n* = 12; MS GG *n* = 13; MS AA *n* = 13). PBMCs: percentage of difference between means in Control GG vs. Control AA = 24.94 ± 20.29%; Control GG vs. MS GG = 29.66 ± 22.75%; Percentage of difference between means in CD3^+^CD20^−^: Control GG vs. Control AA = 43.51 ± 15.54%; Percentage of difference between means in CD3^−^CD20^+^: Control GG vs. MS GG = 59.58 ± 23.05%. (**B**) Endocytosis of A488-BSA at 25 µg/mL for 4 h in the same cellular subsets (controls GG *n* = 15; controls AA *n* = 15; MS GG *n* = 14; MS AA *n* = 12). PBMCs: percentage of difference between means in Control GG vs. MS GG = 32.33 ± 11.69%; CD3^+^CD20^−^: percentage of difference between means in Control GG vs. MS GG = 28.5 ± 11.3%; Percentage of difference between means in CD3^−^CD20^−^: Control GG vs. MS GG = 46.97 ± 17.15%; MS GG vs. MS AA = 38.33 ± 18.73%. Box and whiskers diagrams: the line represents the median value, the box the 25 and 75 percentiles and whiskers mark maximum and minimum values. All comparisons were performed with Student’s *t* test.

Lysosomes are key actors of the endocytic pathway, playing a major role in the degradation of the internalized cargo. Considering the role of MANBA in lysosomal dysfunction, we examined the possible impact of the studied *MANBA* polymorphism on the endocytic function. We evaluated endocytosis with fluorescence-labeled Dextran 10 KDa, a marker of general fluid-phase endocytosis [30], and with serum albumin, which marks receptor-mediated endocytosis [31]. Upon PHA activation in overall PBMCs, a similar pattern of induction for both receptor-mediated and general fluid-phase endocytosis was shared by patients and controls carriers of the AA genotype. However, PBMCs from rs7665090*GG controls showed significantly increased endocytosis compared to those of MS patients with this genotype. Although this significant difference was not reached in B-cells, it was reproduced in both T and NK subsets (Figure 2A,B).

### 3.3. The SNP rs7665090 Modulates Lymphocyte Activation

**Figure 3:**
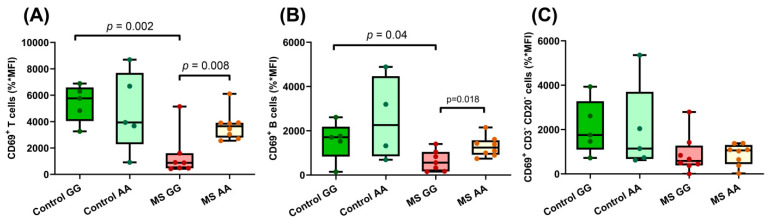
Immune cell activation upon PHA stimulation regulated by rs7665090. The effect of rs7665090 genotypes in CD69 surface expression of (**A**) CD3^+^CD20^−^ lymphocytes (controls GG *n* = 5; controls AA *n* = 5; MSGG *n* = 7; MS AA *n* = 8). Percentage of difference between means in Control GG vs. MS GG = 73.82 ± 17.21%; MS GG vs. MS AA = 61.65 ± 19.8%; (**B**) CD3^−^CD20^+^lymphocytes (controls GG *n* = 5; controls AA *n* = 4; MS GG *n* = 7; MS AA *n* = 8). Percentage of difference between means in Control GG vs. MS GG = 58.62 ± 25.35%; MS GG vs. MS AA = 50.26 ± 18.63%; and (**C**) CD3^−^CD20^−^lymphocytes (controls GG *n* = 5; controls AA *n* = 5; MS GG *n* = 8; MS AA *n* = 8). MFI (median fluorescence intensity) × % of CD69^+^ cells ± standard deviation is shown. Box and whiskers diagrams: the line represents the median value, the box the 25 and 75 percentiles and whiskers mark maximum and minimum values. All comparisons were performed with Student’s *t* test.

We tested whether the allelic change in this MS-risk polymorphism next to the *MANBA* gene, rs7665090, was associated with lymphocyte activation. One of the most studied activation cell markers is the membrane receptor CD69, which is rapidly induced after lymphocyte stimulation. Upon stimulation of PBMCs with PHA, CD69 surface expression was measured in CD3^+^CD20^−^ (T cells), CD3^−^CD20^+^ (B cells), and CD3^−^CD20^−^ (mostly NK) cells. Activation in T and B subsets was significantly downregulated in rs7665090*GG MS patients compared to controls with the same genotype and in rs7665090*AA MS patients (Figure 3A,B). In NK cells, lower levels of activation were observed in MS patients compared to controls (*p* = 0.047), but a genotype-dependent effect was not detected (Figure 3C).

**Figure 4:**
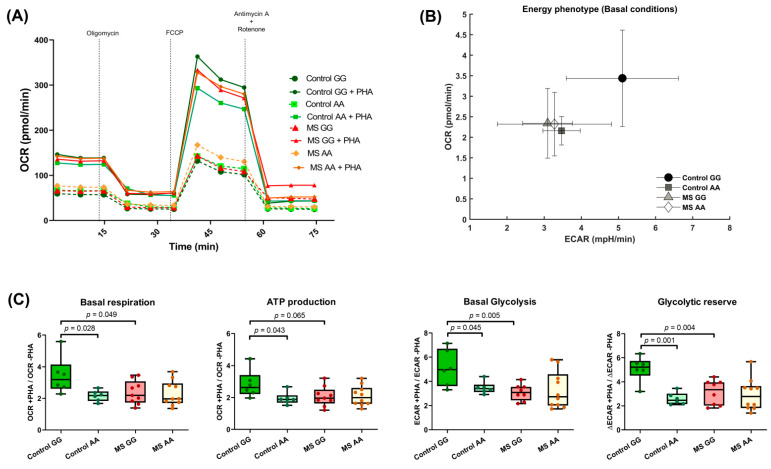
Influence of rs7665090 genotypes on PBMCs metabolism. (**A**) Oxygen consumption rate (OCR) profile of PBMCs from controls and MS patients with/without PHA stimulation stratified by rs7665090 genotype. (**B**) Effect of rs7665090 genotypes on metabolic changes induced by PHA stimulation of PBMCs. An increment of basal respiration induced by PHA over an increment of basal glycolysis (Extracellular acidification rate, ECAR) is shown. (**C**) Effect of rs7665090 on basal respiration, ATP production, basal glycolysis, and glycolytic reserve (controls GG *n* = 6; controls AA *n* = 6; MS GG *n* = 9; MS AA *n* = 10). Basal respiration: percentage of difference between means in control GG vs. Control AA = 37.23 ± 14.53%; control GG vs. MS GG = 31.01 ± 14.28%; ATP production: percentage of difference in between means Control GG vs. control AA = 31.63 ± 13.67%; Control GG vs. MS GG = 27.14 ± 13.42%; Basal glycolysis: percentage of difference between means in Control GG vs. Control AA = 32.08 ± 12.74%; Control GG vs. MS GG = 39.42 ± 11.65%; Glycolytic reserve: percentage of difference between means in Control GG vs. Control AA = 49.06 ± 8.1%; Control GG vs. MS GG = 38.61 ± 11.05%. Box and whiskers diagrams: the line represents the median value, the box the 25 and 75 percentiles, and whiskers mark maximum and minimum values. All comparisons were performed with Student’s *t* test.

A consequence of T-cell stimulation is the shift in cell metabolism from a quiescent to a more energetic state. Under stimulated conditions, glycolytic processes and aerobic respiration are increased to supply the higher energetic requirements [32], and such changes can be measured in vivo with Seahorse technology (Figure 4A). The metabolic shift in PBMCs induced upon PHA stimulation, presented as the ratio between PHA-stimulated and resting conditions, was found more efficient in rs7665090*GG than in rs7665090*AA controls (Figure 4B). In contrast, all MS patients grouped together, showing a mild induction of both aerobic respiration (basal respiration and ATP production) and glycolytic parameters (basal glycolysis and glycolytic spare capacity, Figure 4C).

## 4. Discussion

Translating GWAS signals into functionally relevant results is a challenge. Often, an extensive linkage disequilibrium (LD) surrounding the associated polymorphisms hampers the localization of the causal variant(s). Moreover, the assumption of one causal variant does not fit reality in many regulatory regions, where multiple tightly linked etiological SNPs could be underlying expression quantitative trait loci (eQTL) and GWAS loci, as recently reported [33]. The MS-risk SNP rs7665090 is located in the 3′ end of the *MANBA* gene and included in an LD block together with other 95 proxies (R^2^ > 0.8). Interestingly, all these SNPs are located in *MANBA* intronic or untranslated regions. A recent study in primary biliary cholangitis [34], another autoimmune disease, confirmed the existence of two different LD blocks in this region: one regulating *NFĸB* and represented by rs17032850, and another including *MANBA* and represented by rs227361. This latter SNP (in high LD with rs7665090, R^2^ = 0.81) has been associated with MS risk in a recent GWAS, and both rs227361 and rs7665090 hold the best regulatory scores in the *MANBA* block according to RegulomeDB [35] (Table 1). The Roadmap Epigenomics Project (roadmapepigenomics.org, last accessed 1 June 2022) locates rs7665090 in the transition from a weak to an active enhancer flank, with the overlapping presence of H3K27ac and high levels of H3K4me1, and two MIRb retrotransposon sequences located bordering the SNP. The H3K4me1 signal is associated with prime and active enhancers and may act as a platform for chromatin remodelers [36,37], and the H3K27ac peak also suggests that this region may be a prime enhancer [38]. MIR retrotransposon sequences provide regulatory elements that aid in the organization of chromatin via enhancer-blocking and chromatin barrier activity [39]. In this regard, a recent study on attention deficit hyperactivity disorder showed that rs1054037, a perfect proxy of rs7665090, upregulates *MANBA* expression by disrupting the binding site of hsa-mir-5591-3p [22]. All this epigenetic information supports the possible etiologic role of rs7665090 and some other proxies in a combinatorial mode of multiple causal variants to convey the phenotypic effect of this MS risk locus.

The expression results for the *MANBA* gene in our present study showed significantly decreased levels in lymphocytes from MS patients compared to those from controls. The MS risk SNP, rs7665090, was originally pointed as an eQTL for the *MANBA* gene in blood samples of healthy controls [40]. However, according to the Genotype-Tissue Expression (GTEx) database, the eQTL effect of rs7665090 in whole blood samples only shows a non-significant trend, although this SNP displayed changes in *MANBA* expression differently depending on the tissue (Supplementary Figure S3). In concordance, we did not find an influence of rs7665090 genotypes on *MANBA* expression in PBMCs of controls or MS patients (Figure 1C). As the majority of SNPs identified by GWAS map to non-coding regulatory regions, the underlying functional variants most likely exert their effects only in certain tissues. In parallel to the mRNA expression results, when β-mannosidase activity was measured, decreased levels were found in lymphocytes from patients compared to those from controls. In this case, the MS-risk SNP rs7665090 modified MANBA enzymatic activity, with a significantly higher activity observed in PBMCs from rs7665090*GG controls compared to those from MS patients with the same genotype. As rs7665090 is located in the 3′UTR of the gene, these changes in MANBA activity most probably reflect changes at protein levels, not an altered protein conformation.

Lysosomes are intracellular compartments important for the degradation of biomolecules. The main disaccharide accumulated at lysosomes on account of negligent β-mannosidase activity is Manβ1-4GlcNAc, which has been recently described to possess immunogenic properties [41]. Manβ1-4GlcNAc seems to affect directly myeloid cells and indirectly perturb the humoral response. In fact, mutant mice carrying a truncated form of TREX, a protein implicated in the anabolism of glycans, develop an accumulation of free oligosaccharides and serologic autoimmunity [42]. Thus, we hypothesize that the statistically significant decrease in β-mannosidase activity observed in MS patients homozygous for rs7665090*G (Figure 1D) could induce an accumulation of Manβ1-4GlcNAc, which might affect the immune response. This β-mannosidase enzymatic deficiency correlates with the observed lysosomal dysfunction in this subgroup of patients. In contrast, similar intermediate and apparently tolerated levels of Manβ1-4GlcNAc would be found in MS patients and healthy controls homozygous for rs7665090*A. Therefore, the increased levels of Manβ1-4GlcNAc over a threshold could explain the association between rs7665090 in the *MANBA* gene and MS susceptibility, with a specific impact on patients carriers of the rs7665090*GG genotype.

Moreover, a less efficient lymphocyte activation following PHA stimulation was also detected in MS patients homozygous for the variant rs7665090*G in both T and B cells (Figure 3A,B). This impaired lymphocyte activation is emphasized when some metabolic parameters are considered: a significantly lower basal respiration and ATP production after lymphocyte stimulation and also lower basal glycolysis and glycolytic reserve were found in PBMCs of rs7665090*GG patients compared with those of controls with the same genotype (Figure 4C), indicative of a disrupted mitochondrial function in this subgroup of patients. In contrast, PBMCs of those patients homozygous for the rs7665090*A allele perform almost identical to controls with this genotype. As reported, in some cases, a specific risk variant not only displays *cis* effects, but it may also act in *trans*, even in *loci* mapping to different chromosomes [43]. This could explain why the rs7665090*AA genotype would render a similar behavior in cells of MS patients and controls, while PBMCs of patients carriers of the homozygous rs7665090*GG genotype differ from those of controls, probably due to signals trans-activated through this genotype. This hypothesis agrees with the model of inheritance of complex diseases involving gene regulatory networks.

## 5. Conclusions

This study highlights the importance of the intracellular environment in regulating immune tolerance in MS. We provide evidence regarding the *MANBA* gene as a contributor to MS susceptibility by investigating its role in the immunopathology of MS as well as the impact of the MS-associated SNP rs7665090. Lower *MANBA* expression and enzymatic activity were found in lymphocytes of MS patients compared to those of controls. Concerning rs7665090, we describe how this genetic variant distinctly influences immune cell function. Significantly decreased enzymatic activity and lysosomal function and significantly lower lymphocyte and metabolic activations in response to stimulus were detected in MS carriers of the GG genotype compared to controls with the same genotype, while the rs7665090*AA genotype led to similar effects both in patients and controls. Our approach complements other multilayered studies and delivers further advancement in the molecular events that contribute to MS pathogenesis.

## Figures and Tables

**Table 1:** Analysis of rs7665090 (highlighted in bold letters) and nearby proxies according to RegulomeDB data. 1b: Likely to affect binding and linked to expression of a gene target (eQTL + TF binding + any motif + DNase Footprint + DNase peak); 1f: Likely to affect binding and linked to expression of a gene target (eQTL + TF binding/DNase peak).

SNP	Location	Alleles	Distance to rs7665090 (bp)	D′	R^2^	Correlated Alleles	RegulomeDB	
							Rank	Score
**rs7665090**	**chr4:103551603**	**(A/G)**	**0**	**1.0**	**1.0**	**A = A, G = G**	**1f**	**0.880**
rs4013	chr4:103552813	(C/T)	1210	1.0	1.0	A = C, G = T	1f	0.553
rs735403	chr4:103553543	(C/T)	1940	1.0	1.0	A = C, G = T	1f	0.360
rs735404	chr4:103553665	(G/A)	2062	0.996	0.988	A = G, G = A	1f	0.223
rs2125211	chr4:103559876	(A/G)	8273	1.0	1.0	A = A, G = G	1f	0.223
rs227361	chr4:103586977	(C/T)	35,374	0.93	0.812	A = T, G = C	1b	0.995

## Data Availability

The data that support the findings of this study are available on request from the corresponding author.

## Supplementary Materials

The following supporting information can be downloaded at: https://www.mdpi.com/article/10.3390/ijms23158116/s1.
