# Supplementary figures and images for: Impact of Multiple Sclerosis Risk Polymorphism rs7665090 on MANBA Activity, Lysosomal Endocytosis, and Lymphocyte Activation
Source: Int J Mol Sci. 2022 Jul 23;23(15):8116. doi: 10.3390/ijms23158116 (PMC9331056; doi:10.3390/ijms23158116)

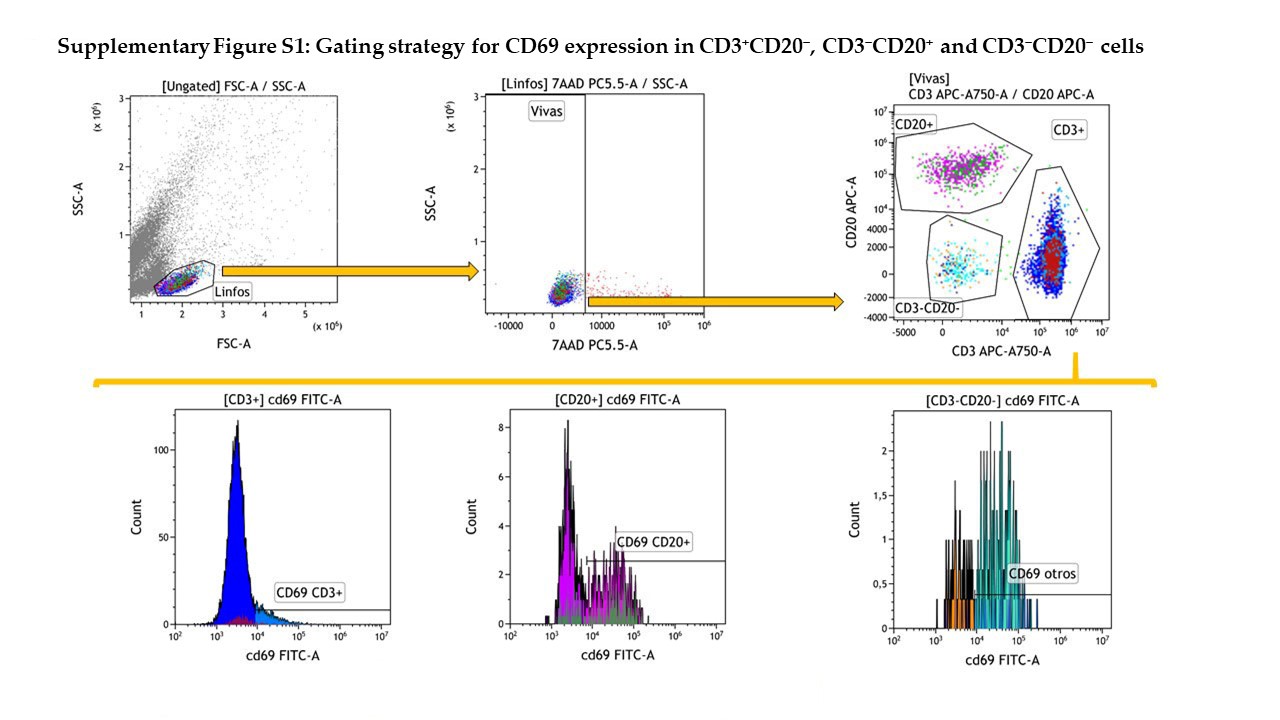

Supplement: Supplementary file 1 [file ijms-23-08116-s001.zip › Supplementary Figure S1.jpg]

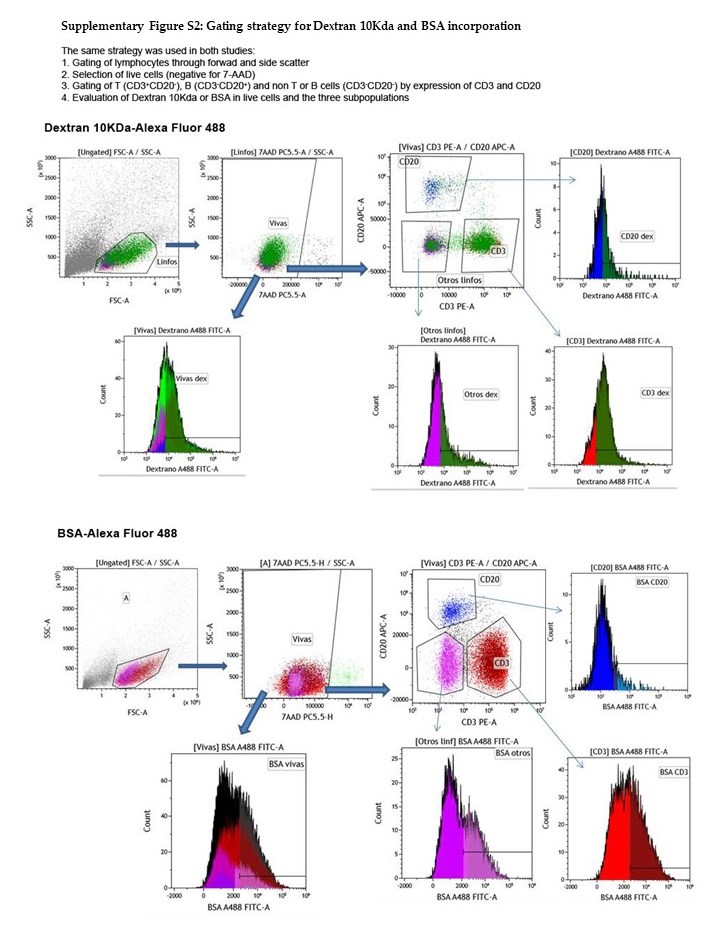

Supplement: Supplementary file 1 [file ijms-23-08116-s001.zip › Supplementary Figure S2.jpg]

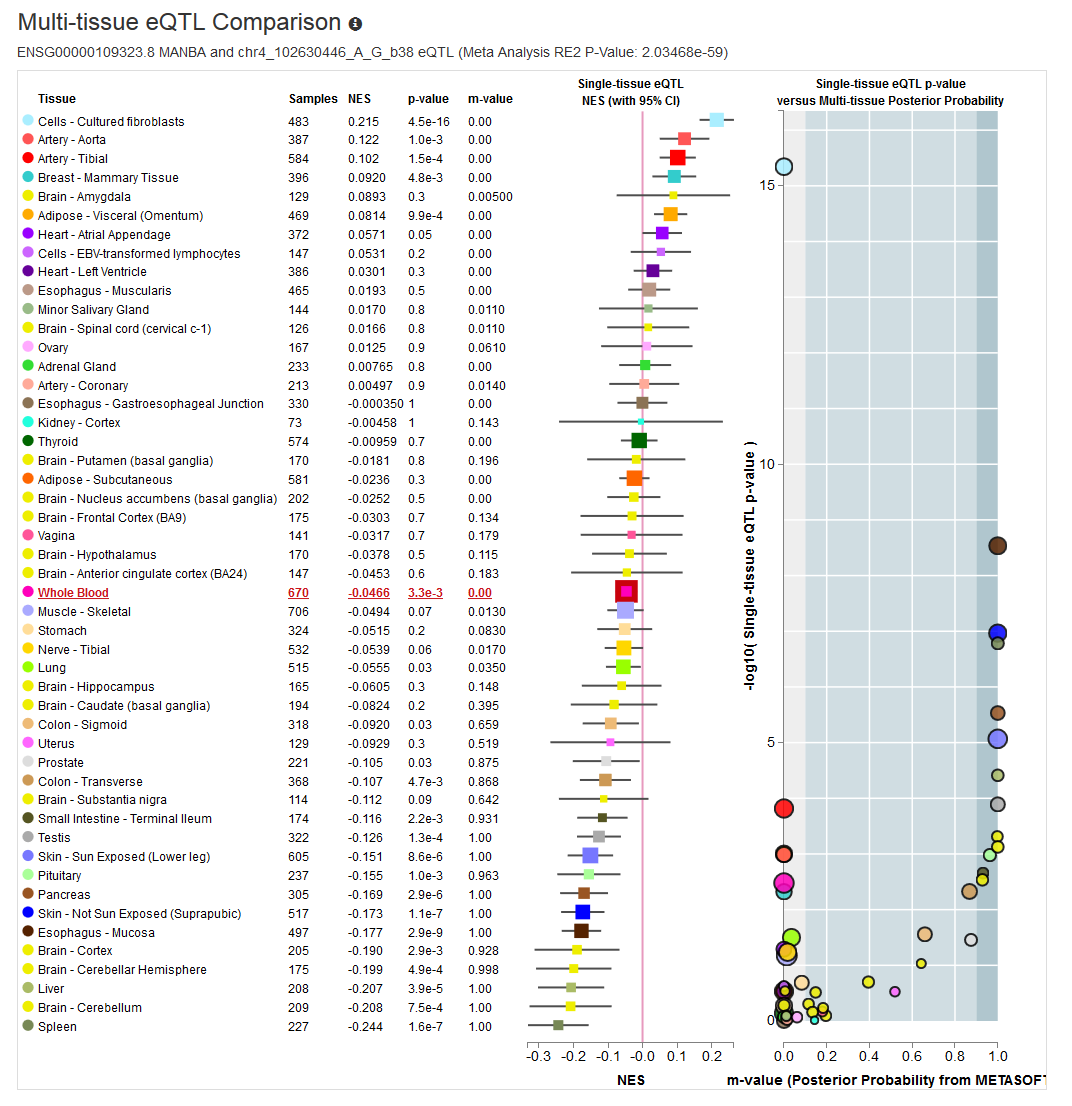

Supplement: Supplementary file 1 [file ijms-23-08116-s001.zip › Supplementary Figure S3.tif]
